# Association between physical activity and activity space in different farming seasons among rural Lao PDR residents

**DOI:** 10.1186/s41182-021-00364-6

**Published:** 2021-09-16

**Authors:** Hongwei Jiang, Lin Lin, Daniel Anthony Yonto, Tiengkham Pongvongsa, Sengchanh Kounnavong, Kazuhiko Moji

**Affiliations:** 1 Research Institute for Humanity and Nature, 457-4 Motoyama, Kamigamo, Kita-ku, Kyoto, 603-8047 Japan; 2 Department of Urban Planning and Design, Xi’an Jiaotong - Liverpool University, Suzhou, Jiangsu Province China; 3 Department of Geography, The University of Alabama, Tuscaloosa, AL USA; 4 Savannakhet Provincial Health Department, Savannakhet city, Savannakhet Province Lao PDR; 5 Lao Tropical and Public Health Institution, Vientiane, Lao PDR; 6 School of Tropical Medicine and Global Health, Nagasaki University, Nagasaki City, Japan

**Keywords:** Physical activity, Activity space, Rural Lao PDR, GPS and accelerometer, Farming seasons

## Abstract

**Background:**

Southeast Asia is experiencing a health transition, where non-communicable diseases (NCD) are exceeding communicable diseases. Despite NCDs accounting for roughly 60–85% of deaths in the region, many developing Southeast Asian countries are beginning to address the impacts of a physically inactive lifestyle for the first time. Our study aims to bridge this gap by objectively measuring physical activity in rural Lao PDR to reveal the association among physical activity, activity space, and seasonal variation.

**Methods:**

Multiple waves of survey data were collected in Songkhon District, Lao PDR between March 2010 and March 2011. Adults aged between 18 and 65 were recruited (*n* = 48). A portable GPS recorded participants’ activity and farmland locations and an accelerometer recorded participants’ physical activity level and daily steps for seven consecutive days. Using a directional distribution tool in ArcGIS 10.5, the activity space area of each participant in each wave was calculated. Concurrently, participants recorded time spent on each daytime activity. Linear mixed models with the fixed effects as the observations from different waves and the random effects as individual participants were developed to identify factors associated with areas of activity space and counts of daily steps, respectively.

**Results:**

A total of 48 respondents aged between 19 and 57 took part in the study. Half of the participants were females. Walking was found to be the most frequent travel mode. Females were physically less active, with a smaller activity space, and were more overweight than the males in the study. Participants were physically less active during the off-farming seasons.

**Conclusions:**

Findings contribute to the surveillance of risk factors needed to create healthy living environments. Our research is also one of the first to use empirical evidence demonstrating seasonal variations of rural residents’ activities in mainland Southeast Asia.

**Supplementary Information:**

The online version contains supplementary material available at 10.1186/s41182-021-00364-6.

## Background

Southeast Asia is experiencing a health transition, where non-communicable diseases (NCD) are exceeding communicable diseases [1]. In 2016, NCDs accounted for roughly 60–85% of deaths across the region [1, 2], with physical inactivity responsible for approximately 3.2% of the disease burden [3]. One of the main challenges associated with physical inactivity is that it decreases energy expenditure, which increases the epidemic of overweight and obesity [3–5]. A physically inactive lifestyle also leads to higher morbidity rates from cardiovascular disease, ischemic stroke, and metabolic syndrome [3, 6]. Although physical inactivity is a common concern in developed countries [7], many developing countries in Southeast Asia are beginning to note the impacts of a physically inactive lifestyle for the first time [8] as overweight and obesity concerns start to rise [2, 9–11].

One of the primary data collection instruments in social, health, and epidemiological research is the survey questionnaire [12]. Using subjective methods, such as the International Physical Activity Questionnaire (IPAQ) or Global Physical Activity Questionnaire (GPAQ), these surveys assess the physical activity level of the population in developing countries [5, 13, 14]. However, objective measures using pedometers or accelerometers provide a more precise and valid measures of time spent in various physical activities [15–18]. Objective measures are used less often in developing countries due to financial constraints or a lack of expertise managing the technology [19, 20]. Thus, the lack of objective physical activity data from developing countries is a significant barrier in advocating public health interventions with cultural and environmental sensitivity to improve physical activity in chronic disease prevention.

Activity space can be quantified through several measures [21–27]. Several transportation studies used data from the time–space diary technique; however, this requires a high degree of commitment and collaboration with participants [28, 29], which makes replicating these studies in rural Southeast Asia challenging due to the subsistence lifestyles of its residents. Compared to traditional travel diaries and questionnaires, global positioning system (GPS) tracking devices enhance the ability and reliability of collecting large amounts of trajectory data. As a result, the overall data quality has been greatly improved. At the same time, activity space measured by GPS and step count by accelerometer/pedometer has been increasingly used in physical activity studies to examine the associations between environments and activity [30, 31]. The spaces within which people actually move and are exposed to could explain inter- and intra-personal variations in spatial habits [30]. Hence, understanding activity space could help explain different levels of physical activity.

Environmental factors are expected to be related to physical activity in multiple life domains: leisure/recreation/exercise, occupation, transportation, and household [32]. To identify the association between environment or activity space and physical activity, considerable research has been conducted through a GPS and a geographic information system merged with accelerometer data [33–36]. Although most activity space research is conducted in developed countries, developing countries provide a unique set of challenges that remain understudied [37]. Developing countries in Southeast Asia differ in their built environment, economic activities, seasonal changes, and cultural norms from developed countries in the west [38]. Thus, solutions aimed at increasing physical activity in developed countries, such as parks or sidewalks, may not be effective or directly applicable to developing countries [39].

To this end, our study uses GPS and accelerometer devices to objectively measure physical activity in rural Lao PDR. In doing so, we aim to reveal the association among physical activity, activity space, and seasonal variation in this understudied region. Our results contribute to the surveillance of risk factors needed to create environments that are conducive to healthy living in Southeast Asia and baseline information of activity changes in rural Laos [2].

## Methods

### Study area

**Fig. 1:**
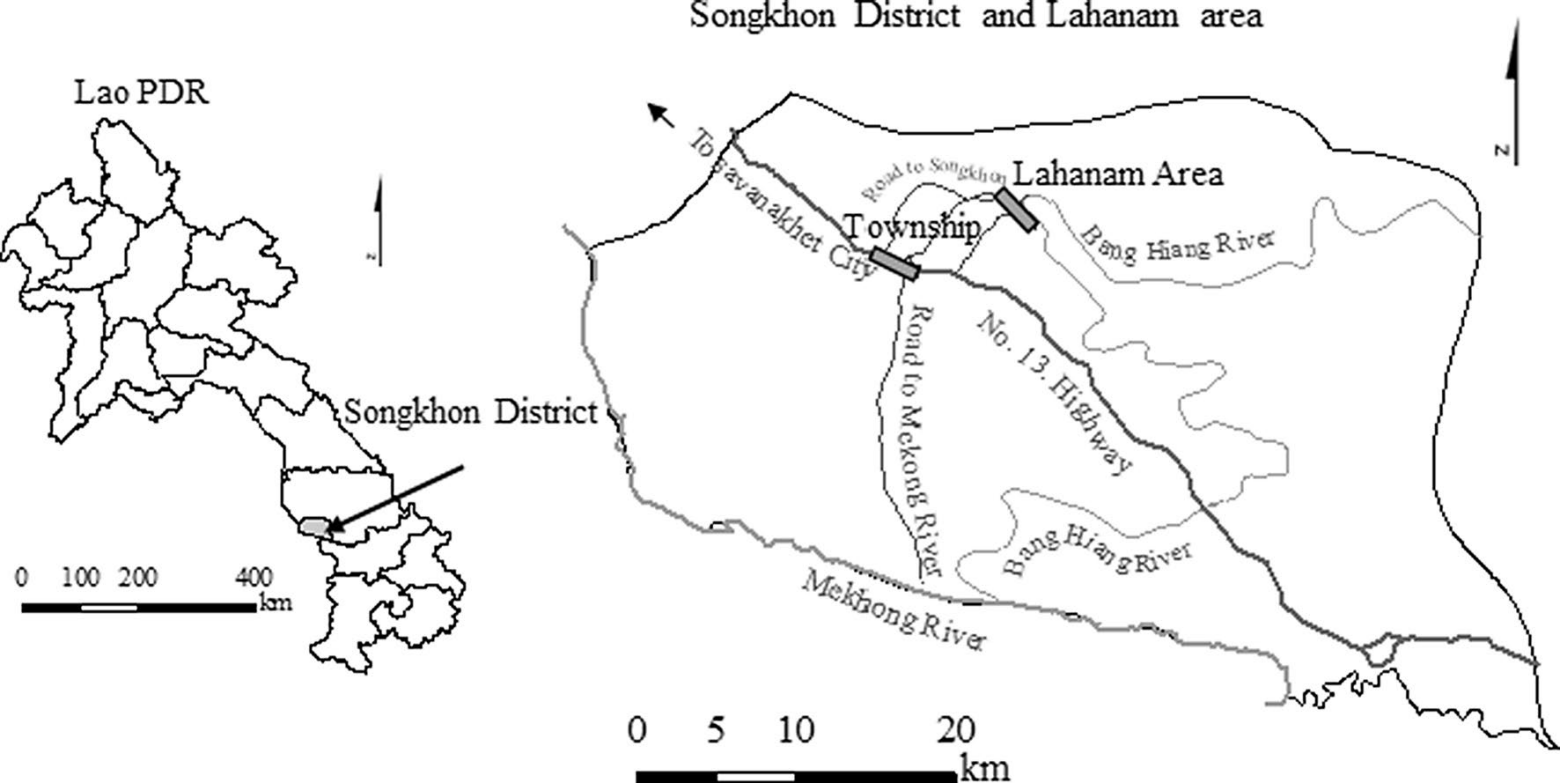
Lahanam area in Songkhon District, Lao PDR. The target village, located inside the rectangular shape labeled as Lahanam Area on the map, is situated along the west bank of the Bang Hiang River (This map was created by the authors)

This study was conducted in Songkhon District, Savannaket Province, Lao PDR (Fig. 1), located near Lahanam, which is situated along the Bang Hiang River, a branch of the Mekong River. The climate is considered tropical monsoon, where the wet season lasts from April to October. The average seasonal temperature is about 27 °C with an average rainfall of roughly 1390 mm during the wet season. The dry season starts from November and ends in March with an average seasonal temperature of 23 °C and an average precipitation of roughly 73 mm (Unpublished records of Savannakhet Provincial Meteorological Section). The village has a total population of 1091 registered residents, with 628 between 18 and 65 years as of March 1, 2010 (Village Chief notes). The actual adults who lived in the village fluctuated during the time of our study due to about 30% of the population participating in seasonal work in Thailand and Vientiane (capital of Lao PDR).

A typical family in Lahanam earns a living by rice cultivating, livestock grazing, fishing, and cloth weaving. In general, livestock grazing is the work of male adults and weaving is the work of female adults (Unpublished observations). However, both female and male adults engage in fishing year-round (Unpublished observation). Consistent with other regions of Lao PDR, rice cultivation is the major subsistence of local residents [40].

Our study area has roughly four farming seasons: wet farming, wet off-farming, dry farming, and dry off-farming. Two types of rice cultivation are carried out in the wet and dry seasons. The wet season rice cultivation, which is rain-fed, starts from May and is harvested in October [40]. The dry season cultivation is carried out in sections of the rice paddy with irrigation from December to April. The drastic differences in precipitation between wet and dry seasons have a direct impact on individual activities. To that end, our survey is predicated on the four seasons based around Lahanam’s farming activities.

### Data collection

To capture the seasonal activity changes of local residents, multiple waves of surveys were carried out in Lahanam from June 2010 to March 2011. In each wave, two devices were used to collect data for each participant for seven consecutive days. The first, a portable GPS device (M241, Holux Technology, Inc.), recorded activity and farmland location of participants. The second, an accelerometer instrument (Lifecorder EX, Suzuken Ltd.), measured the participant's physical activity level and steps. Recording was made every twelve seconds for the GPS and 2 min for the accelerometer. Concurrently, a self-reported log by each participant detailed the time spent on each activity from 6 am to 6 pm. The body weight and height of each participant was measured in each wave by the researchers. GPS data were validated by the accelerometer and, if no record existed in the accelerometer, or if GPS data did not agree with accelerometer data, we excluded the data.

To test the GPS devices battery and the waterproofness of the device shell in the study area, 20 participants, 10 males and 10 females aged between 18 and 65 years, were recruited by convenience sampling through the village head in March 2010. The formal data collection started from June 2010. Due to the availability of the devices, about 30 able-bodied adults aged between 18 and 65 (pregnant women were excluded as well) were recruited each wave from Wave 1 to Wave 4. Not all participants completed all four waves to covered four different farming seasons, namely, wet farming season (Wave 1: June 2010), wet off-farming season (Wave 2: September 2010), dry farming season (Wave 3: December 2010) and dry off-farming season (Wave 4: March 2011). Twenty thousand Lao kips (USD $2.50) was given to each participant who completed one wave, with a total of 100,000 Lao kips (USD $12.5) given to participants who completed all five survey waves. Wave 1 to Wave 4 were included in the further analyses.

Neighborhood built environment attributes were derived from google maps and QuickBird satellite image which was taken on January 9, 2008. Euclidean distances from participant’s home to different types of land uses, such as paddy, the health center, the primary school, the middle school, the local market, the temple, and the mini shop in the village were measured to identify different destinations, where residents frequently visited.

### Creating activity space

**Fig. 2:**
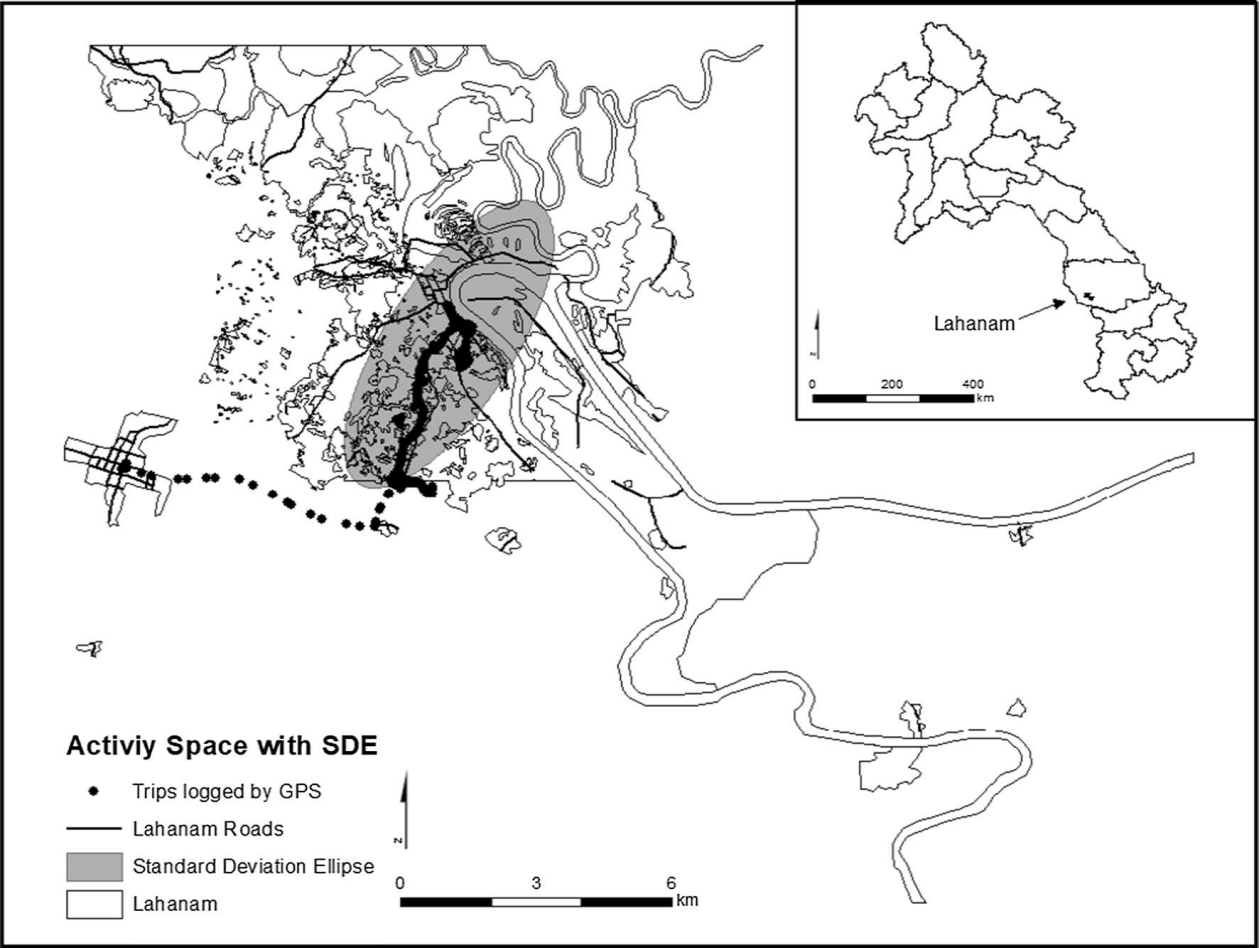
Example of a participant’s daily activity movement with a 2-standard deviation ellipse representing the participant’s activity space shaded in gray (This map was created by the authors)

The most common method to operationalize activity space is the standard deviation ellipse (SDE) that measures the directional distribution of a series of GPS points or the “densest” areas, where most of the individual mobility occurs [41]. In this study, for each participant in each wave, activity space was delineated as an ellipse centered at the home and extended to two standard deviations of the observed activity locations that were recorded by the GPS device worn by each participant (see Fig. 2 for a participant’s 1-day activity). The area of each activity space was calculated using a directional distribution tool in ArcGIS 10.5. The tool summarized spatial characteristics and created an SDE polygon as the output activity space. A 1-, 2-, and 3-SDE polygon was computed to cover 68%, 95%, and 99% of the input features, respectively. Considering that a 1-SDE covers 68% of activity points, several participants’ activity space may not be constructed accurately using the 1-SDE approach. Conversely, using a 3-SDE may include outliers that distort the shape of the ellipse and introduce concerns that could result in measuring the extreme extent of travel and capture large geographic areas that are not visited by an individual. Therefore, our study used a 2-SDE polygon to delineate activity space in line with previous research [42].

### Statistical analysis

Bivariate analysis of variables derived from the daytime activity log, and distance measures of land use were carried out with areas of activity space and counts of daily steps. Furthermore, ANOVA tests controlling for different waves were conducted for the two dependent variables by gender, and the self-reported time for different activities by gender by wave, respectively. Linear mixed models with the fixed effects as the observations from different waves and the random effects as individual participants were developed to identify factors associated with areas of activity space and counts of daily steps, respectively. Maximum likelihood (ML) and restricted maximum likelihood (REML) estimation were applied in estimating variance and covariance parameters in the linear mixed models. SPSS software (version 22.0) was used for all statistical analysis.

## Results

### Descriptive statistics

Table 1 summarizes participant’s individual characteristics, daytime activity log, and distance from participant’s home to different destinations in the village. A total of 48 participants between 19 and 57 years took part in the four waves of the study. Half of the participants [24] were females. The average age of the participants was 36.^1^ The average body mass index (BMI) of participants was 22.7 kg/m^2^. One-fourth of the female participants were overweight with BMIs more than 25 kg/m^2^, and one eighth of male participants were overweight. On average, 31 participants took part in each wave. Only six participants finished all four waves, while 22 and 13 participants did three and two waves of four waves, respectively, and seven participants one wave only.

**Table 1:** Summary of participant’s individual characteristics, daytime activity log, and distance from participant’s home to different destinations in the village

Category			Wave 1 (Jun 2010)				Wave 2 (Sep 2010)				Wave 3 (Dec 2010)				Wave 4 (Mar 2011)			
			M		F		M		F		M		F		M		F	
			Mean	SD	Mean	SD	Mean	SD	Mean	SD	Mean	SD	Mean	SD	Mean	SD	Mean	SD
Individual characteristics	Number of Participants		15		15		16		16		13		19		14		15	
	Age		39.6	10.0	36.8	9.9	37.3	9.8	35.1	10.0	38.0	10.7	35.4	10.2	36.5	8.6	35.9	7.8
	Body weight (kg)		55.5	7.9	54.3	12.5	58.6	9.8	51.3	6.9	57.4	10.1	52.1	9.6	58.5	9.7	55.6	11.4
	Body height (cm)		158	5	151	9	161	6	151	5	159	6	151	7	161	6	152	8
	BMI (kg/m^2^)		22.0	2.3	23.6	3.8	22.6	2.8	22.4	2.5	22.4	2.5	22.9	3.0	22.5	2.9	24.0	3.7
	Number of Overweight and obesity (BMI ≥ 25 kg/m^2^)		2		5		3		2		2		5		2		5	
Daytime activity log (hours)	Time spent for outdoor activities	Rice cultivating	4.4	1.9	2.0	1.9	2.1	2.0	0.8	1.0	3.8	2.9	2.0	2.3	1.6	1.6	0.7	0.8
		Fishing	0.6	1.0	0.0	0.0	0.5	1.2	0.0	0.0	0.1	0.2	0.0	0.1	0.6	0.7	0.0	0.1
		Livestock grazing	0.9	1.0	0.9	0.9	2.0	2.3	0.6	0.8	0.8	0.9	0.5	0.6	1.8	1.5	0.8	1.0
		Hunting & gathering	0.1	0.2	0.4	0.5	0.1	0.3	0.2	0.3	0.1	0.3	0.2	0.7	0.2	0.3	0.2	0.5
	Time spent for indoor activities	Weaving	0.0	0.0	1.6	1.9	0.0	0.0	2.6	2.3	0.0	0.0	1.8	2.2	0.0	0.0	2.4	2.2
		Housework	0.5	0.8	2.6	2.2	0.6	0.8	3.2	1.6	1.7	1.7	2.7	1.8	2.9	2.2	2.9	2.5
	Time spent for other activities	Chatting with other people, eating and drinking, and resting	5.5	1.6	4.4	1.0	6.6	2.1	4.6	1.3	5.4	1.6	4.7	1.0	5.0	1.5	5.0	1.5
Distance from participant’s home to different destinations (meters)	Paddy field^a^		1724	879	1724	878	1699	1233	1698	1233	1882	1429	1875	1138	1610	1237	1509	12,252
	Health center		483	79	482	78	562	262	563	262	551	304	539	249	552	288	554	273
	Primary school		539	77	538	76	617	266	618	266	606	308	595	252	608	292	609	277
	Middle school		415	78	414	76	493	266	494	266	482	308	471	252	484	292	485	277
	Local market		415	79	415	79	495	258	496	257	484	299	471	245	485	283	487	268
	Temple		319	70	319	69	341	122	340	122	374	133	344	119	361	130	353	121
	Mini shop		68	55	68	53	124	238	124	237	160	259	121	218	139	254	128	245

^a^Two participants who did not have paddy fields were excluded from the calculation of distance from participants’ home to paddy fields

Among all participants, two participants did not have farmland. The Euclidean distances of the remaining participants’ farmland to their homes ranged between 65 and 4808 m, with an average of 1729 m. The average distances from participant’s home to health center, primary school, middle school, local market, temple, and mini shop are 532 m, 588 m, 464 m, 465 m, 337 m, and 109 m, respectively.

The average sizes of the activity space were 29.6 km^2^, 16.9 km^2^, 9.5 km^2^, and 37.9 km^2^ for wet farming season (Wave 1), wet off-farming season (Wave 2), dry farming season (Wave 3), and dry off-farming season (Wave 4), respectively. The average daily steps for Waves 1, 2, 3, and 4 are 16,861, 13,205, 14,736, and 13,297, respectively. There were three female participants with average daily steps fewer than 10,000 steps—a conventional threshold for inactive lifestyle [43, 44]—in Wave 1, five females and two males with average daily steps fewer than 10,000 steps in Wave 2, and four females in Wave 3 and six females in Wave 4 with average daily steps fewer than 10,000. Table 2 summarizes the size of activity space and counts of daily steps by gender for each wave.

**Table 2:** Activity space and daily steps by gender and by wave

Wave		Female		Male	
		Mean	SD	Mean	SD
Wave 1 (Jun 2010)	Activity area^a^	6.9	8.8	52.3	125.8
	Steps^b^	12.9	2.9	20.8	5.8
	No. of persons	15		15	
Wave 2 (Sep 2010)	Activity area Area (km^2^)area (km^2^)	8.6	21.1	25.2	42.0
	Steps	11.0	3.5	15.5	5.3
	N of persons	16		16	
Wave 3 (Dec 2010)	Activity area Area (km^2^) area (km^2^)	6.4	6.4	14.1	10.1
	Steps	12.4	3.1	18.2	3.9
	N of persons	19		13	
Wave 4 (Mar 2011)	Activity area Area (km^2^)area (km^2^)	16.0	30.9	61.4	155.8
	Steps	11.2	4.1	15.5	4.3
	N of persons	15		14	

^a^Measured in km^2^

^b^Measured in 10^3^ steps/day

### Bivariate analysis

To compare activity space and total steps between female and male, natural logarithm transformation was carried out for the area of each activity space and each participant’s counts of daily steps. Table 3 summarizes the natural logarithm transformation of areas of activity space and total counts of steps by wave and by gender and ANOVA test results. ANOVA tests showed that the area of activity space is significantly different between females and males for Waves 1 and 4. The counts of daily steps are significantly different between females and males throughout all waves. ANOVA tests were also conducted for self-reported time spent on different activities by gender for each wave. Table 4 summarizes the ANOVA results. Male participants spent significantly more time on outdoor activities than the female participants in all four waves. The former spent more time than the latter for other activities, such as chatting with other people, eating and drinking, and resting in Waves 1 and 2. Meanwhile, female participants spent significantly more time on indoor activities, such as weaving and doing housework in all four waves.

**Table 3:** ANOVA results of activity space and total steps by gender and by wave

Wave		Female		Male		ANOVA
		Mean	SD	Mean	SD	*p* value
Wave 1 (Jun 2010)	LN (2SD SQM)^a^	0.75	2.03	2.33	1.75	**0.030**
	LN (daily steps)^b^	9.44	0.23	9.90	0.30	**0.001**
Wave 2 (Sep 2010)	LN (2SD SQM)	− 0.08	2.16	1.42	2.21	*0.062*
	LN (daily steps)	9.24	0.38	9.58	0.42	**0.026**
Wave 3 (Dec 2010)	LN (2SD SQM)	0.66	2.22	1.97	1.82	*0.090*
	LN (daily steps)	9.40	0.25	9.78	0.24	**0.001**
Wave 4 (Mar 2011)	LN (2SD SQM)	− 0.19	2.95	2.33	1.69	**0.010**
	LN (daily steps)	9.26	0.38	9.62	0.27	**0.007**

*P* value < 0.1 are shown italic

*P* value < 0.05 are shown bold

^a^LN (2SD SQM): natural log transformation of activity space

^b^LN (daily steps): natural log transformation of counts of daily steps

**Table 4:** ANOVA results for self-reported time for different activities between 6 am and 6 pm by gender by wave (h/day)

Wave	Activity	Female		Male		ANOVA
		Mean	SD	Mean	SD	*p* value
Wave 1	Outdoor activities	3.4	2.3	6.0	1.9	**0.002**
	Indoor activities	4.2	2.3	0.5	0.8	**0.001**
	Other activities	4.4	1.0	5.5	1.6	**0.027**
Wave 2	Outdoor activities	1.6	1.4	4.8	2.1	**0.001**
	Indoor activities	5.8	2.0	0.6	0.8	**0.001**
	Other activities	4.6	1.3	6.6	2.1	**0.003**
Wave 3	Outdoor activities	2.7	2.1	4.9	2.5	**0.013**
	Indoor activities	4.5	2.4	1.7	1.7	**0.001**
	Other activities	4.7	1.0	5.4	1.6	0.149
Wave 4	Outdoor activities	1.7	1.2	4.2	2.3	**0.001**
	Indoor activities	5.3	2.1	2.9	2.2	**0.005**
	Other activities	5.0	1.5	5.0	1.5	0.953

*P* value < 0.05 are shown bold

Table 5 summarizes results of the Pearson’s correlation analysis. The area of activity space and counts of daily steps were positively significantly correlated. The *r* of 0.539 is moderate to strong correlation with a high statistical significance (*p* < 0.01). In addition, time spent on indoor activities such as weaving and doing housework was negatively significantly associated with daily steps and activity space. An *r* of − 0.395 is weak to moderate correlation with a high statistical significance (*p* < 0.01). Time spent on outdoor activities such as cultivating rice, fishing, livestock grazing, and hunting and gathering was also positively significantly associated with daily steps and activity space. The *r* of 0.282 is weak correlation with a high statistical significance (*p* < 0.01) (Additional file 1: Table S1).

**Table 5:** Pearson correlation results

	Daily steps (Natural log transformed)	Activity space (Natural log transformed)
Activity space (natural log transformed)	0.539***	
Time spent on indoor activities (weaving and doing housework)	− 0.518***	− 0.395***
Time spent on outdoor activities (fishing, livestock grazing, and hunting and gathering)	0.353***	0.282***
Time spent on other activities (chatting with others, eating and drinking, and resting)	− 0.137	0.147
Time spent on rice cultivating	0.490***	0.186**
Distance from home to paddy	− 0.030	0.060

***p value < 0.01

**p value < 0.05

### Regression model results

Based on the bivariate analysis results, two linear mixed models were developed for daily steps and activity space, respectively. The fixed effects included observations of participant’s characteristics such as age and gender, time spent on outdoor activities, and distance from home to participant’s paddy field. Wave information was also added into the model as a category variable using Wave 4 as the reference. The individual participants were included as the random effect to control for variations of individual participants. Table 6 summarizes the linear mixed model results. Females were found to have significantly fewer steps and smaller activity space compared with those of their male counterparts. Age was negatively significantly associated with activity space only. As expected, time spent on outdoor activities was positively significantly associated with steps and activity space. Compared with Wave 4, participants in Waves 1 have significantly more daily steps, and participants in Wave 3 showed a positive marginal significance. No significant difference was found for activity space among different waves.

**Table 6:** Linear mixed model results (fixed effect)—daily steps and activity space

	Daily steps (Natural log-transformed)		Activity space (Natural log-transformed)	
	*B*	*p* value	*B*	*p* value
(Constant)	9.262	0.000	2.104	**0.015**
Gender (male vs. female)	0.172	**0.009**	1.368	**0.004**
Age	− 0.003	0.306	− 0.069	**0.002**
Outdoor^a^ activity time	0.081	**0.000**	0.194	*0.052*
Distance^b^ from home to paddy (km)	− 0.043	***0.092***	0.130	0.455
Wave 1	0.142	**0.030**	0.295	0.587
Wave 2	− 0.003	0.962	− 0.443	0.398
Wave 3	0.113	***0.054***	0.068	0.894
Wave 4^c^	0.000			

*P* value < 0.1 are shown bold and italic

*P* value < 0.05 are shown bold

^a^Time spent on outdoor activities, such as rice cultivating, fishing, livestock grazing, and hunting and gathering

^b^Distance from home to paddy field in km

^c^Reference category

## Discussion

Our findings showed that participants’ daily steps and activity space were positively significantly associated with each other. In other words, with more daily steps, a participant’s activity space increased. Despite some study participants owning motorcycles, the substandard road conditions required many participants to walk while performing daily activities. Examples include rice cultivating, paddy fishing, livestock grazing, and individual hunting and gathering. These activities, measured by step count, are also consistent with our field observations. Taken together, these results imply that our study participants were more likely to travel on foot for their daily activities. This is quite different from studies in the U.S. and China, where driving and car ownership are associated with larger activity space [41, 45]. These findings underscore the importance of identifying place-based solutions to public health challenges [39], especially in developing Southeast Asian countries.

Although study participants traveled mostly by foot, female participants were found to have significantly fewer daily steps and smaller activity spaces than those of the male participants. This finding is similar to the findings in other countries, where an increasing gender gap of physical activity has been observed [5, 46–48]. Our study also found that one-fourth of the female participants and one-eighth of male participants were overweight with BMIs greater than 25 kg/m^2^. Despite finding lower overweight rates among male participants, these results were consistent with a LAO PDR STEPS report conducted by the World Health Organization [49]. Our findings suggest that analyses of activity space would be able to evaluate risks of obesity in paddy farming communities.

The division of labor in our study area may be the core issue of this finding. For instance, male participants were found to spend much more time on outdoor activities including rice cultivating, fishing, and livestock grazing. Female participants, on the other hand, spent the majority of their time on indoor activities, such as weaving and housework. Our findings are consistent with the previous studies on labor division in Lao PDR [50]. Surprisingly, despite walking being the most frequent travel mode in the study area, there are still a few participants with daily steps fewer than 10,000, an important indicator for an active lifestyle [43, 44]. One explanation, as shown in Table 4, is that both male and female participants spent more than one-third of their day on sedentary or low-level physical activities, such as chatting, resting, drinking or eating. However, females spent more time on low-level physical activity indoors, such as weaving and housework. Therefore, with gyms, parks, and other public spaces not readily available in the region, it is difficult for rural Southeast Asians to increase physical activity in a subsistence lifestyle. Thus, future studies and intervention programs could focus on how to incorporate physical activities into leisure time to promote healthier lifestyles, particularly for rural Southeast Asian females, as males tend to receive more physical activity from their work in the region.

Activity space was not found to have significant seasonal variations. On the other hand, when comparing physical activities of different farming seasons, participants in Wave 1 (wet farming season) and Wave 3 (dry farming season) were found to have more daily steps than those participants in Wave 4 (dry off-farming season). These findings indicated that village residents have their routine destinations and physical activity levels of residents in the region are strongly impacted by farming. In other words, frequency of visiting those routine destinations show seasonal variations, with farming seasons having higher frequent visits than dry off-farming season. This evidence demonstrates that seasonal variation does impact rural resident activities in mainland Southeast Asia. Thus, future interventions aimed at increasing physical activity should prioritize the off-farming season, where subsistence work is reduced and residents are less physically active.

Our study also has a number of limitations. For instance, the sample size is relatively small. Moreover, our study only collected data for 1 year. Longitudinal studies with historical data and larger sample sizes would bring richer insights into the health transition of developing countries. In addition, our sample represented “active” farmers rather than the “inactive” industrialized urban people, who are more at risk of obesity, in Southeast Asia. Finally, our study does not address the caloric intake of participants. As overweight and obesity are generally caused by the imbalance between calorie consumption and intake, this acts as an important distinction to address. However, our results are a useful baseline to physical activity and activity space of farmers in Southeast Asia.

## Conclusion

Our study is one of the first to objectively measure activity space and physical activity to assess the impact of farming seasons in rural Southeast Asia. Physical activity levels are strongly impacted by whether residents farm or not in the region. Integrating GPS tracking and accelerometer data to depict an activity space, where individuals travel and how much time they spent on different daily activities allows for advances in assessing the health risk factors of both communicable and non-communicable diseases. Understanding daily travel activities in this way may contribute to effective intervention programs needed to create healthy living environments [51, 52]. On the one hand, it improves the awareness for the necessity of physical activity. On the other, it creates a method to provide affordable equipment to assess physical activity with limited public health resources in Southeast Asia.

## Supplementary Information

**Additional file 1.** ANOVA results of activity space and total steps by age category and by wave.

## Data Availability

The data sets used and/or analyzed during the current study are available from the corresponding author on reasonable request.

## Abbreviations

BMI: Body Mass Index
NCD: Non-communicable disease
GPS: Global poisoning system
IPAQ: International Physical Activity Questionnaire
GPAQ: Global Physical Activity Questionnaire
SDE: Standard deviation ellipse

## Footnotes

1 Due to the lack of the Census data at the rural villages in Laos, we carried out our own population count with the help of the village head in the study area before the formal data collection started. Comparing the sampled residents and the residents aged between 18 and 65 who did not include in our survey, no significant differences were shown in terms of age (36.2 ± 9.6 vs. 35.0 ± 13.1, t (547) = -0.557, p = 0.557) and the sex ratio (50% vs. 46.9%, χ^2^(1) = 0.168, p = 0.682).
