# Supplementary material for: Association between physical activity and activity space in different farming seasons among rural Lao PDR residents
Source: Trop Med Health. 2021 Sep 16;49:73. doi: 10.1186/s41182-021-00364-6 (PMC8444593; doi:10.1186/s41182-021-00364-6)
Supplement: Supplementary file 1 — Additional file 1. ANOVA results of activity space and total steps by age category and by wave. [file 41182_2021_364_MOESM1_ESM.docx]

Supplementary Table 1

| **Wave** |  | **18-39 years old** | |  | **40 years and over** | |  | **ANOVA** |  |
| --- | --- | --- | --- | --- | --- | --- | --- | --- | --- |
|  |  | *Mean* | *SD* |  | *Mean* | *SD* |  | p-value |  |
| Wave 1  (Jun 2010) | LN (2SD SQM)^1^ | 1.90 | 1.82 |  | 1.18 | 2.20 |  | 0.337 |  |
|  | LN (daily steps)^2^ | 9.69 | 0.37 |  | 9.65 | 0.34 |  | 0.785 |  |
|  | N of Persons | 15 | |  | 15 | |  |  |  |
| Wave 2  (Sep 2010) | LN (2SD SQM) | 0.89 | 2.44 |  | 0.42 | 2.15 |  | 0.568 |  |
|  | LN (daily steps) | 9.35 | 0.46 |  | 9.47 | 0.40 |  | 0.447 |  |
|  | N of Persons | 17 | |  | 15 | |  |  |  |
| Wave 3  (Dec 2010) | LN (2SD SQM) | 1.30 | 2.00 |  | 1.05 | 2.37 |  | 0.748 |  |
|  | LN (daily steps) | 9.55 | 0.33 |  | 9.56 | 0.29 |  | 0.935 |  |
|  | N of Persons | 18 | |  | 14 | |  |  |  |
| Wave 4  (Mar 2011) | LN (2SD SQM) | 1.49 | 3.22 |  | 0.45 | 1.85 |  | 0.312 |  |
|  | LN (daily steps) | 9.48 | 0.34 |  | 9.38 | 0.42 |  | 0.490 |  |
|  | N of Persons | 16 | |  | 13 | |  |  |  |
